# Animate monitoring is not uniform: implications for the animate monitoring hypothesis

**DOI:** 10.3389/fpsyg.2023.1146248

**Published:** 2023-04-27

**Authors:** Jeff Loucks, Berit Reise, Rosselle Gahite, Shaun Fleming

**Affiliations:** ^1^University of Regina, Regina, SK, Canada; ^2^Universität Osnabrück, Osnabrück, Germany

**Keywords:** animate monitoring, attention, animacy, perception, visual search, inattentional blindness

## Abstract

The animate monitoring hypothesis (AMH) purports that humans evolved specialized mechanisms that prioritize attention to animates over inanimates. Importantly, the hypothesis emphasizes that any animate—an entity that can move on its own—should take priority in attention. While many experiments have found general support for this hypothesis, there have yet been no systematic investigations into whether the type of animate matters for animate monitoring. In the present research we addressed this issue across three experiments. In Experiment 1, participants (*N* = 53) searched for an animate or inanimate entity in a search task, and the animate was either a mammal or a non-mammal (e.g., bird, reptile, insect). Mammals were found significantly faster than inanimates, replicating the basic AMH finding. However, they were also found significantly faster than non-mammals, who were not found faster than inanimates. Two additional experiments were conducted to probe for differences among types of non-mammals using an inattentional blindness task. Experiment 2 (*N* = 171) compared detection of mammals, insects, and inanimates, and Experiment 3 (*N* = 174) compared birds and herpetofauna (reptiles and amphibians). In Experiment 2, mammals were spontaneously detected at significantly higher rates than insects, who were detected at only slightly higher rates than the inanimates. Furthermore, when participants did not consciously identify the target, they nonetheless could correctly guess the higher level category of the target (living vs. nonliving thing) for the mammals and the inanimates, but could not do so for the insects. We also found in Experiment 3 that reptiles and birds were spontaneously detected at rates similar to the mammals, but like insects they were not identified as living things at rates greater than chance when they were not consciously detected. These results do not support a strong claim that all animates are prioritized in attention, but they do call for a more nuanced view. As such, they open a new window into the nature of animate monitoring, which have implications for theories of its origin.

## 1. Introduction

For a great majority of animals across a wide variety of ecosystems, those that pay attention to other animals in their immediate environment would seemingly survive longer. New et al. (2007) proposed the animate monitoring hypothesis (AMH), which states that humans (and potentially other animals) are biologically predisposed to pay greater attention to animates over inanimates, as a result of our evolution (and which is also likely shared with nearby evolutionary cousins). Using a change detection paradigm, they found that people detected changes to animate entities in scenes more quickly and more frequently than changes to inanimate entities.

This hypothesis has garnered significant interest, and numerous additional studies have provided support for the AMH. Animates are also detected more quickly in visual search tasks (Lipp et al., 2004; Jackson and Calvillo, 2013), more frequently reported in attentional blink and inattentional blindness tasks (Calvillo and Jackson, 2014; Guerrero and Calvillo, 2016), and receive longer fixations (Yang et al., 2012). Not every investigation has found support, however. Notably, Hagen and Laeng (2016) found no animate advantage in change blindness once the visual context of the scene had been accounted for. Hagen and Laeng (2017) also found that animates do not induce or reduce attentional blinks, though they are more accurately reported in such tasks (see also Hagen et al., 2018). Avoiding such visual confounds, Loucks et al. (2020) demonstrated that 4-year-old children remember a novel, arbitrary sequence of actions better if the sequence contains an animate rather than inanimate entity, despite identical appearances. Taken together with other complementary findings (Pratt et al., 2010; Nairne et al., 2017; van Buren and Scholl, 2017; Nguyen and van Buren, in press), these results suggest that once an entity has been ascribed an animate status, heightened attention and cognition follows.

While some of the above research indicates that animates stimuli are in general prioritized in cognition, little attention has been paid to the specific animate used. Importantly, the AMH would not suggest that there should be any difference according to the type of animate; an evolved system such as this should prioritize any animate in the observer’s immediate vicinity. However, in another experiment of Loucks et al. (2020), they found that using a toy dog improved children’s memory better than a toy beetle. It is possible that insects may not be considered the same kind of animate as mammals. For instance, most people consider mammals to be more similar to humans than insects (Eddy et al., 1993), and more worthy of moral consideration (Kellert, 1993; Tisdell et al., 2006). The amount of visual experience observers have for each type of animate also likely differs, in addition to the particular quality of that experience (e.g., Knight, 2008). Possidónio et al. (2019) also found that various types of animals differ from one another to observers on the basis of dimensions such as valence, arousal, and dangerousness, which may also affect their cognitive processing more generally.

The difference that Loucks et al. (2020) observed between the dog and the beetle was relatively weak, in a statistical sense, and it has only been observed with children, not adults. But, in considering the possibility that humans might attend to different types of animates differently, it is striking that research on animate monitoring tends to use mammals much more often than other types of animals. Most importantly, no research to date has ever systematically compared different types of animates in terms of their capacity to capture attention (with the exception of snakes: LoBue and DeLoache, 2008; and spiders: New and German, 2015).

Research on the neural correlates of animate processing in adults supports the possibility that the type of animate may matter for animate monitoring. For example, animals appear to be processed in a graded fashion according to perceived animacy (Connolly et al., 2012), and that those judged as being more animate (e.g., humans, chimpanzees, cats) activate distinct regions of lateral occipital cortex (LOC) relative to tools, but that those judged as being less animate (e.g., fish, insects) activate overlapping regions of LOC relative to tools (Sha et al., 2015). Importantly, this concept of perceived animacy goes beyond a binary definition of animacy—the latter would refer only to whether an entity has the capacity for self-initiated movement, while the former involves a graded concept of agency in relation to humans.

However, two other bodies of work suggest that the type of animate should not matter. One is that of Thorpe and colleagues on rapid visual categorization (e.g., Macé et al., 2009; Crouzet et al., 2012; Wu et al., 2015), which has shown that adults can identify animals within approximately 120 ms. Importantly, this rapid detection occurs at the superordinate level of “animal,” and not at the basic level of “dog” or “bird” (Wu et al., 2015). Thus, at early levels of awareness, adults know that there is some kind of animal present, without knowing exactly what animal it is. But as with the literature on the AMH, while a fairly wide range of animal types are used in this line of research, a large proportion are mammals, and no specific comparisons have been made between different types.

Another is the literature on visual features diagnostic of animates vs. inanimates, which are thought to be distinguishable on the basis of mid-level features such as the degree of curvilinearity (Wichmann et al., 2010). Long et al. (2017) created synthetic images of animals and objects that preserved certain texture and form information but removed basic-level diagnostic information: “texforms.” They found that observers could find texforms faster when they were embedded among texforms of a different higher level category (e.g., finding an animal among objects) than when they were from the same category, and that the degree to which a texform displayed curvilinearity was predictive of whether it was classified by observers as an animal (see also Zachariou et al., 2018). However, recently He and Cheung (2019) equated animals and tools in gist statistics—by using elongated and round types of both—and found that observers were still faster at detecting animals. Taken together with some of the findings of Long et al. (2017), it appears that visual features cannot entirely account for the animate advantage. In any case, no systematic comparison between animal types has been made in this literature.

Thus, in the current research, we aimed to compare the attentional capture of (non-human) mammals against a variety of non-mammals. In contrast with the AMH, we hypothesized that mammals would hold a higher status in attention over non-mammals, and would thus be detected more easily/rapidly in a variety of tasks. We believe they hold this elevated status given their higher similarity to humans, either in form or in perceived animacy, and/or the different experiences humans have with them. In terms of the general animate advantage, we hypothesized that mammals would be detected more easily/rapidly than inanimates, but were unsure about non-mammals in this respect. In an initial experiment, we first assessed whether mammals were generally advantaged in detection over a diverse group of non-mammals in visual search. Two additional experiments were then conducted to compare detection of mammals against specific types of non-mammals using inattentional blindness, to extend the results of the first experiment by way of a different methodology.

## 2. Experiment 1

In Experiment 1 we compared search times for mammals against a heterogeneous group of non-mammals in a visual search task. Our primary interest with this first experiment was to determine whether there would be any global advantage for mammals above other animals, rather than comparing them to specific classes at the same level (e.g., reptiles, birds) in an exhaustive sense. We also did not control for all visual features in our entities, such as curvilinearity or gist, as we wanted to have entities be easily recognizable and in their typical posture. However, we did ensure that targets were equated on certain visual features, such as luminance and contrast (e.g., the SHINE toolbox, Willenbockel et al., 2010). We also specifically selected mammals that our (primarily White Canadian) participants had little experience with, due to the fact that there are likely pre-existing differences in exposure to mammals and non-mammals. We predicted that mammals would be detected faster than non-mammals and inanimates, but that non-mammals would not be detected faster than inanimates.

### 2.1. Method

#### 2.1.1. Participants

Participants were 53 University of Regina undergraduate students (8 male), who earned partial course credit for their participation. We aimed for a sample size of 34, as this would allow us to detect a medium effect size (*d* = 0.50; assuming power = 0.80, and *α* = 0.05), but ended up sampling more participants to satisfy student demand in our department. An additional 4 individuals participated (1 male) whose data was dropped because they provided valid reaction time data for less than 50% of trials. Self-reported race of our sample was: White (*n* = 31), South Asian (*n* = 10), Black (*n* = 4), mixed (*n* = 3), East Asian (*n* = 2), Middle Eastern (*n* = 1), and undisclosed (*n* = 1).

#### 2.1.2. Stimuli

Stimuli consisted of greyscale images of 48 animates and 48 inanimates. For both groups of stimuli, 16 were target images, 16 were associated presentation images, and 16 were distractor images. Presentation images were secondary images of the targets, so that only the category of the target was cued on the presentation screen, and not the exact target image to be found on the search screen. For the animates there was a further subdivision of 8 mammals and 8 non-mammals among the targets and presentation images. We specifically wanted to avoid selecting animals that are encountered frequently by Western adults (e.g., dogs, pigs). The 8 mammal targets were an armadillo, a camel, a chinchilla, a ferret, a lemur, an okapi, a rhino, and a saiga. The 8 non-mammal targets were a centipede, a crab, a gecko, a mantis, a puffin, a squid, and a turtle. Our selection of these particular animals was based on a desire to have variety in overall appearance across mammals and non-mammals. The 16 inanimate targets were a baseball glove, a belt, a bottle opener, a watering can, a cassette tape, a drill, an egg slicer, a picture frame, a hole puncher, a blender, a lawnmower, a sled, a speaker, a staple remover, a tea infuser, and a tricycle. Distractor stimuli were other animates and inanimates that never served as targets (and half of the animate distractors were mammals, half non-mammals). We also analyzed the average luminosity and contrast (the standard deviation of the luminance distribution) of all target stimuli using the SHINE toolbox (Willenbockel et al., 2010). As a group, none of the three entity types were different from each other on either of these variables, nor were the animates as a group different from the inanimates, all *t*’s > 1.6, *p*’s > 0.11. The entire set of stimuli can be viewed at: https://osf.io/b2zpf/?view_only=4129537fbef14d018b52e90e6a4c55c1.

#### 2.1.3. Design and procedure

**Figure 1:**
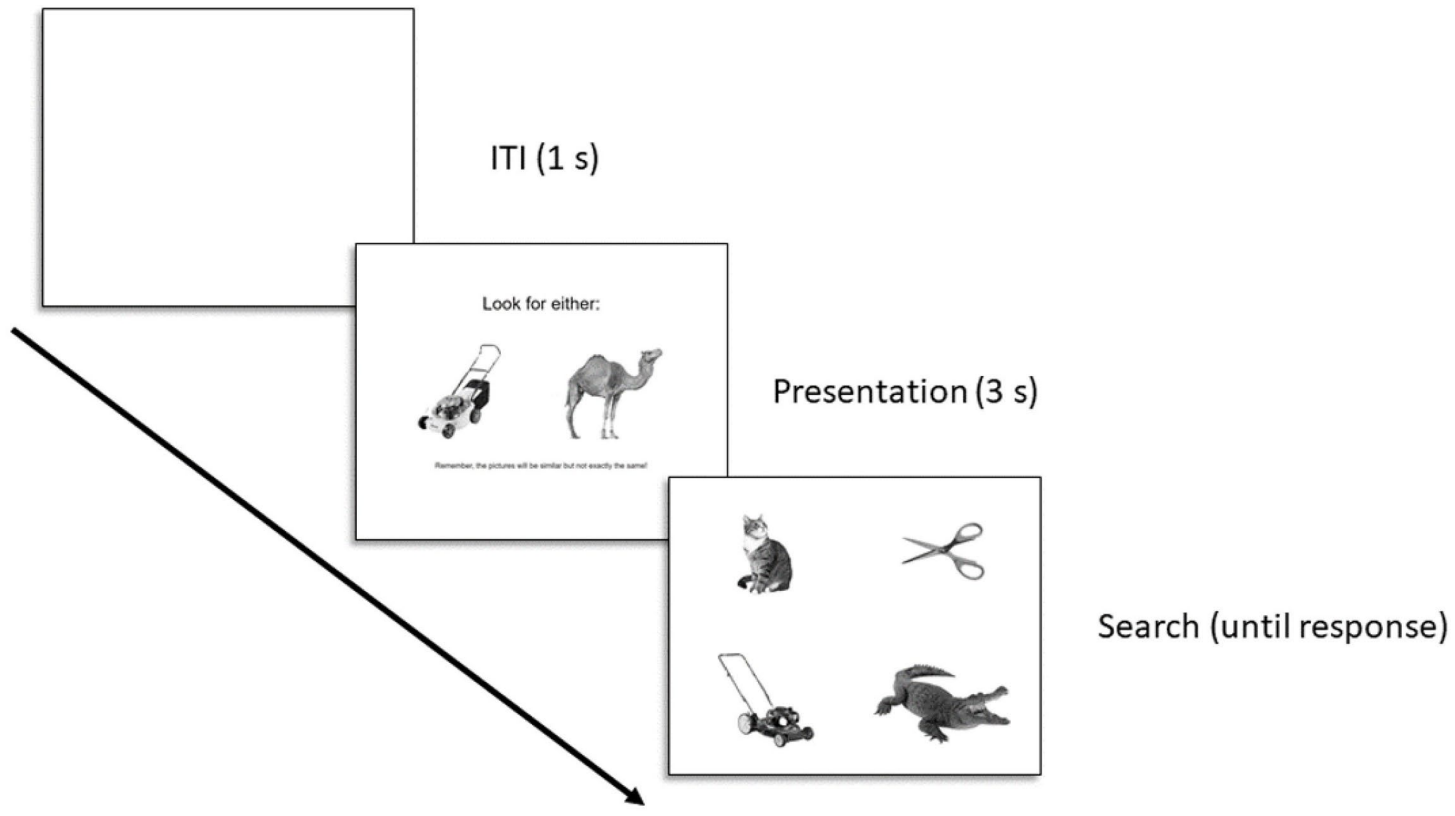
Trial structure for Experiment 1. The text below the images on the presentation display read: “Remember, the pictures will be similar but not exactly the same!”.

Figure 1 displays the trial structure. Each trial consisted of a presentation screen (3 s) and a search screen (until response). The ITI was fixed at 1 s. Presentation screens displayed one animate and one inanimate image as possible targets to look for on the upcoming search screen; the specific images on the presentation screen were different than the target images (see stimuli above). Stimuli were positioned along the center of the y-axis of the screen and at 25 and 75% of the x-axis of the screen. The target on the presentation screen was randomly selected (e.g., squid), and then paired with a random target from the opposing category (e.g., tricycle). The search screen displayed the selected target and three additional distractors, arranged in a 2 × 2 grid. The stimuli were centered within each of the four quadrants defined by bisecting the screen along the x-and y-axes. Regardless of the target category, each search screen displayed two animate and inanimate entities, and the specific positioning of each stimulus within the quadrants was random. Participants were required to indicate which position on the grid contained the target entity, using the T, Y, G, and H keys on their keyboard, as quickly and accurately as they could. The right/left position of each target on the presentation screen was randomly selected. A first block of trials displayed targets in one right/left position on the presentation screen, and then a second block displayed them in the opposite position. Trial order within a block was random, and there was no break between blocks (to the participant it was all one block). Thus each target was searched for two times total, across a total of 64 trials. A video of sample trials can be found at: https://osf.io/b2zpf/?view_only=4129537fbef14d018b52e90e6a4c55c1.

The experiment was implemented in PsychoPy and was hosted on Pavlovia.org. After providing consent, participants were asked to report on their gender and race/ethnicity, and then provided an instruction screen. Participants were told that on each trial, they would be shown an animal and an object, and would have to find either the animal or the object on a subsequent screen, as quickly and accurately as they could.

### 2.2. Results

Accuracy was high: 97.2% correct in the mammal condition, 95.9% correct in the non-mammal condition, and 94.8% in the inanimate condition. Since the task was straightforward for participants in terms of accuracy, this supports the idea that reaction time would be a more sensitive measure of the search efficiency for this task.

**Figure 2:**
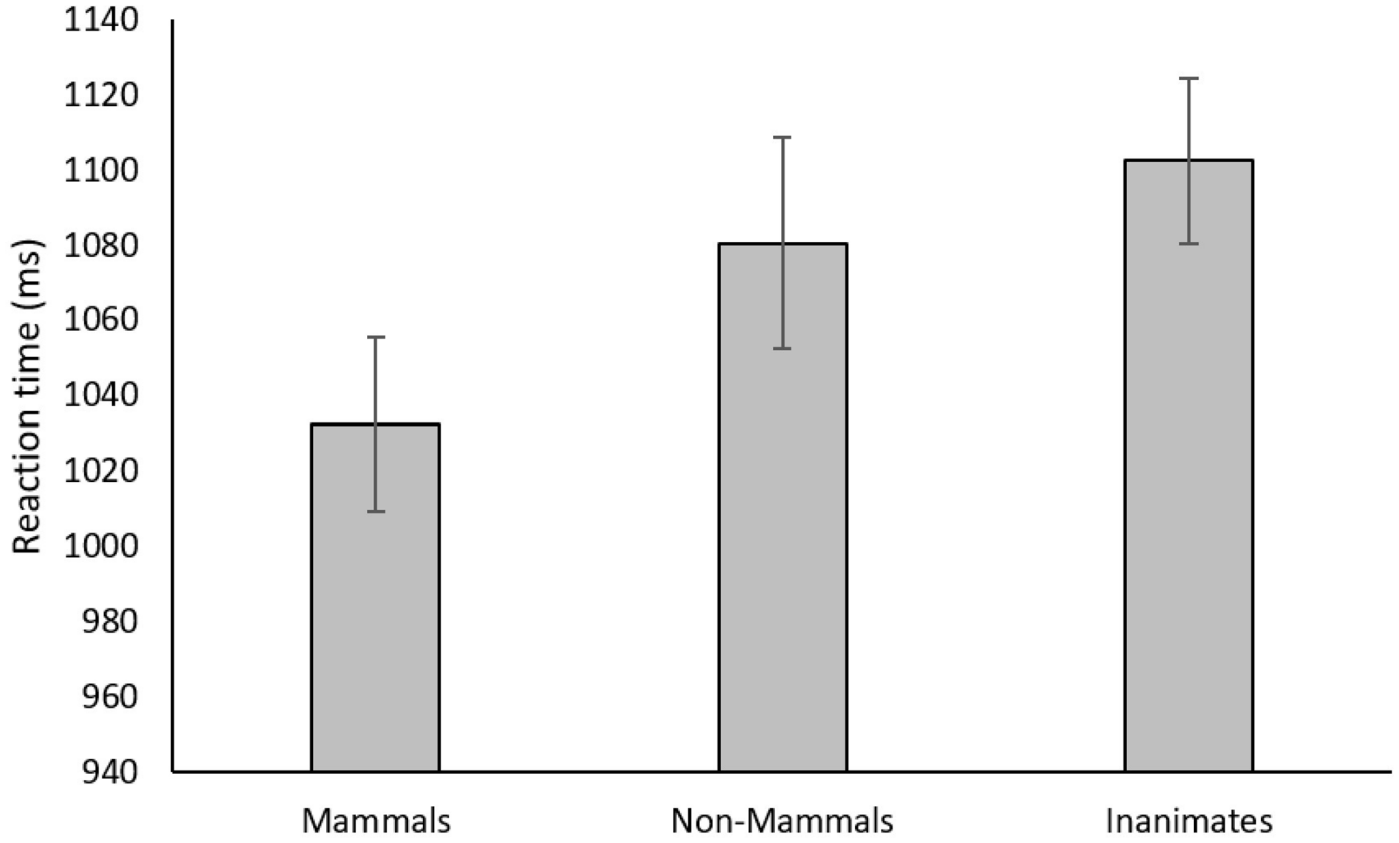
Mean reaction times as a function of entity type in Experiment 1. Error bars represent standard error.

Only reaction times for accurate identifications that occurred within 2,000 ms of the search screen onset were included in the analysis (14.1% of trials excluded). Figure 2 displays these mean reaction times for each type of entity. A repeated measures ANOVA on entity type was significant, *F*(2, 104) = 8.82, *p* < 0.001, partial *η*^2^ = 0.15. Paired samples *t*-tests revealed that search times for mammals were significantly faster than those for non-mammals, *t*(52) = 2.88, *p* = 0.006, Cohen’s *d* = 0.40, and inanimates, *t*(52) = 4.14, *p* < 0.001, *d* = 0.57, and that search times for non-mammals were not different than those for inanimates, *t*(52) = 1.26, *p* = 0.21. A Bayesian analysis of the difference between the non-mammals and the inanimates provided moderate evidence for the null hypothesis, *BF_01_* = 3.18.

**Figure 3:**
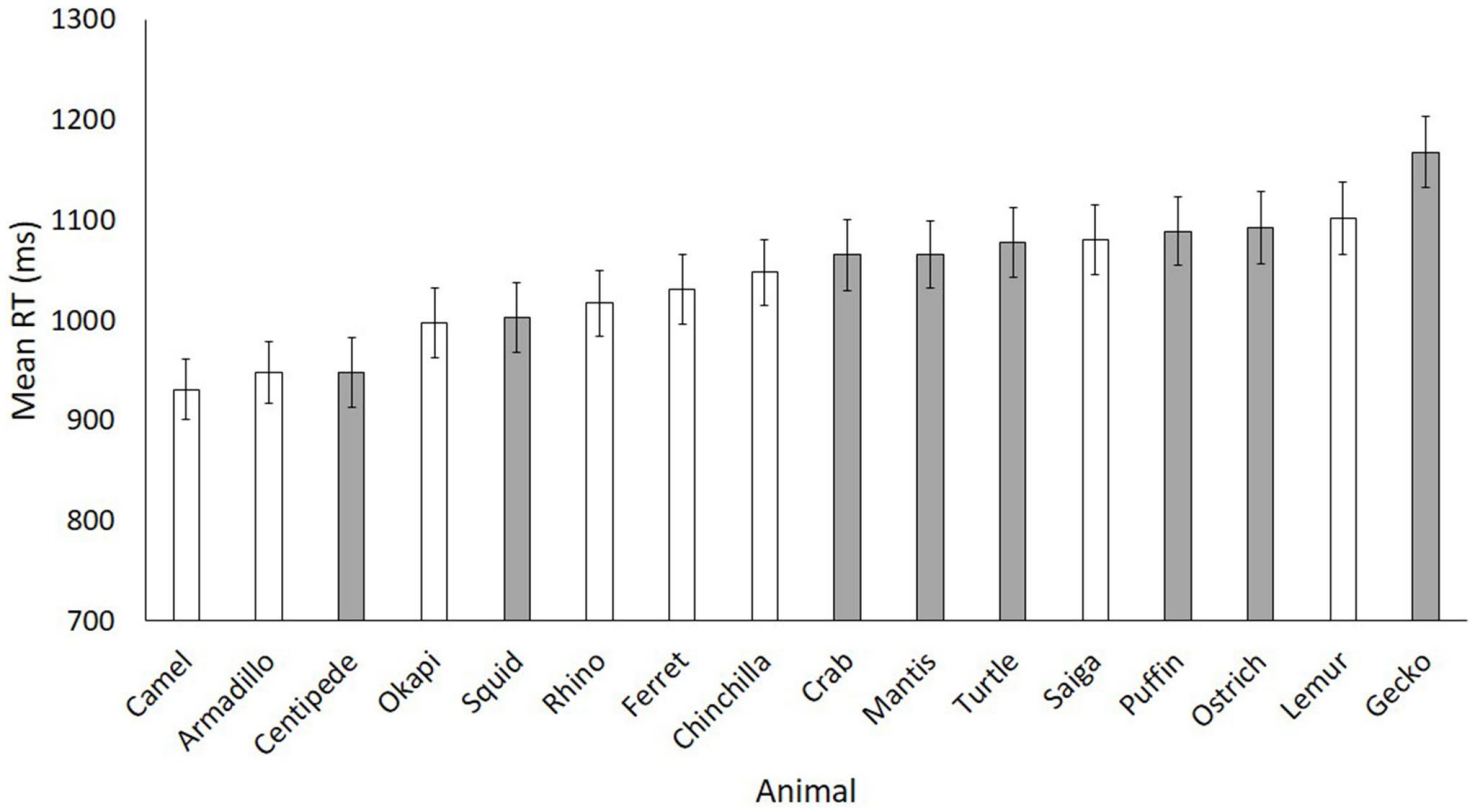
Mean reaction times for each animal in Experiment 1. Mammals are white, non-mammals are grey. Error bars represent standard error.

Figure 3 displays the mean reaction times for each animal in rank order. Although there is a fairly clear separation of the mammal searches being generally faster than the non-mammal searches, it is not perfectly clean: two of the non-mammals are among the faster ranks, and two of the mammals are among the slower ranks. Although matched on luminosity and contrast, we cannot control for all visual differences between our stimuli, and thus we cannot fully determine why an individual animal may have been easier to find than another. However, our main prediction was that, even amidst the noise of different body shapes, faces, patterns, and textures, there would nonetheless be a signal that stands out as the mammal/non-mammal distinction in search times.

### 2.3. Discussion

The results of Experiment 1 indicate that not all animals appear to be equal in their ability to capture observers’ attention: mammals are detected more rapidly than non-mammals in visual search. It is important to note that we selected mammals which are not common in Westerner’s daily experience—in an attempt to put them on more equal footing with the non-mammals—but which were likely easily binned by our participants into the relevant categories (e.g., mammal, bird, insect). Thus, it is not likely a difference in experience with specific targets which drives the effect. Instead, it is likely the detection of perceptual features that are diagnostic of the particular category (e.g., four limbs, furry)—or the combination of more than one feature—which drives the increased fidelity of observer’s attention. Non-mammals were not simply detected more slowly than mammals—importantly, they were also not detected significantly faster than inanimate objects. This result does not support the idea that animate monitoring is based on “pure” animacy—the capacity for internally generated motion. All of our non-mammals have this property, and none of our inanimates do. Although this negative result with non-mammals seems to stand in contrast to a relatively large literature supporting the AMH, recall that most studies of the AMH use primarily mammals as stimuli.

However, strong conclusions should not be drawn from a single experiment. If mammals indeed hold special status over other animals in this respect, then this advantage should also be observed using different methods. Thus, one goal of Experiments 2 and 3 was to replicate this basic advantage using an inattentional blindness task. A secondary goal of these additional experiments was to get more fine-grained data on the relative advantage of mammals over specific classes of animals—specifically, insects, birds, and herpetofauna (reptiles and amphibians).

## 3. Experiment 2

Experiments 2 and 3 are highly similar experiments, but we report them individually because they were collected at different times with slightly different samples. Both utilized an inattentional blindness paradigm to explore how well various animate and inanimate entities capture observers’ attention when they appear unexpectedly amidst another task. In developing this experiment we were inspired by the work Calvillo and Jackson (2014), and utilized a task and stimuli akin to theirs. The stimuli we used were thus normed line drawings from Snodgrass and Vanderwort (1980). These stimuli contrast with those from Experiment 1, where the goal was to select animates that our participants would have less experience with; In this case the stimuli are more familiar and recognizable animals, which is important for conscious verbal reporting of the stimuli when they appear unexpectedly.

In Experiment 2, we first explored whether mammals would be detected more robustly than insects and inanimates. This was achieved by inserting an unexpected image of an entity amidst a secondary, sham task (finding a color word). In addition to the standard data on each participants’ ability to spontaneously detect the unexpected image, we also asked participants to guess if the image that they saw was of a living or non-living thing, regardless of whether they spontaneously noticed the image or not. This measure assessed the ability of participants who failed to consciously recognize the image to nonetheless correctly identify a critical aspect of its identity through unconscious/partially conscious recognition. We predicted that mammals would be detected more readily than insects and inanimates, and that insects would not be detected more readily than inanimates.

### 3.1. Method

#### 3.1.1. Participants

Participants for Experiment 2 were 171 University of Regina undergraduate students (42 male, 2 non-binary), who earned partial course credit for their participation. This sample size was chosen based on a power analysis assuming the same percentage difference as obtained in Calvillo and Jackson (2014) for animates vs. inanimates in their low load condition (~30%). Self-reported race of the sample was: White (*n* = 95), South Asian (*n* = 21), Black (*n* = 11), South-East Asian (*n* = 11), East Asian (*n* = 9), Middle Eastern (*n* = 7), Indigenous (*n* = 7), Métis (*n* = 6), mixed (*n* = 3), and Afro Carribean (*n* = 1). An additional four participants were sampled but dropped, as they did not notice the color word on two or more real trials, with one of these being the critical trial.

Participants were randomly assigned to one of the three target conditions. Approximately equal numbers of participants were assigned to the mammal (*n* = 57), insect (*n* = 54), and inanimate (*n* = 61) conditions.

#### 3.1.2. Stimuli

The target stimuli for the inattentional blindness task were black and white line drawings of five mammals (camel, deer, fox, lion, raccoon), five insects (ant, bee, beetle, butterfly, grasshopper), and five inanimate objects (flag, helicopter, kite, sled, whistle), taken from Snodgrass and Vanderwort (1980). We selected fewer animals than in experiment 1 (from 8 to 5) as we were constrained by the available choices of this particular stimulus set. Our selection of the specific 5 for each category was initially based on our intuitions about how readily participants would be able to name the entities if they did happen to notice them. Following this initial selection, entities were chosen for all categories that had roughly similar scores for familiarity and naming agreement, in the middle range for both measures (Snodgrass and Vanderwort, 1980). Each image measured 300 × 300 pixels. They can be viewed at https://osf.io/b2zpf/?view_only=4129537fbef14d018b52e90e6a4c55c1.

The target stimuli for the sham word finding task were color words. The targets in the practice trials were green, blue, and red, and in the real trials orange, purple, and yellow. This target order was fixed, and thus yellow was the target on the critical (inattentional blindness) trial for all participants. Non-target words were various 3–6 letter words that were non-color and non-animal words. All words were presented in capital letters in black.

#### 3.1.3. Design and procedure

**Figure 4:**
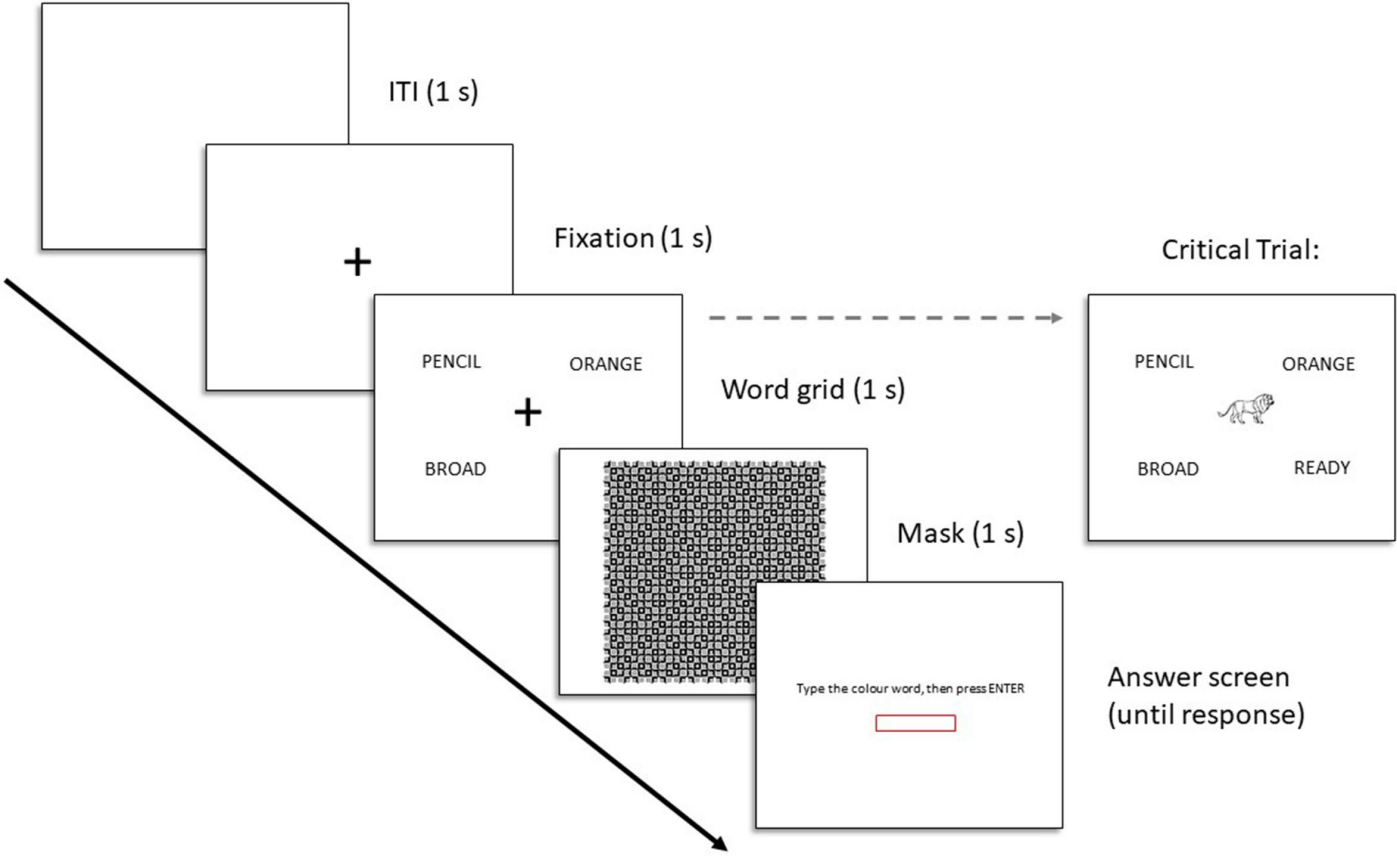
Trial structure for Experiments 2 and 3.

Five of the six trials of the sham task consisted of a blank screen (1 s), a central fixation cross (1 s), a word grid surrounding the fixation cross (1 s), a perceptual mask (1 s), and an answer screen (until response). A schematic of each trial and the critical trial can be found in Figure 4. On the grid screen the words were presented in the northwest, northeast, southwest, and southeast corners of the screen, surrounding the fixation. The specific visual angle of the stimuli was dependent on the participants screen size. The sixth and final trial was the critical trial, which was identical to the first five trials except that the fixation cross on the word grid screen was replaced with one of the target images. Each participant only saw one target on this one critical trial.

The experiment was implemented and hosted using the Gorilla Experiment Builder (www.gorilla.sc; Anwyl-Irvine et al., 2020). After providing consent and demographic information, participants were instructed that in each trial, four words would be quickly flashed on the screen, and their job was to find the one color word amidst this set. Participants completed three practice trials first, during which they received feedback about their performance. All participants were correct on at least 2/3 practice trials. Following practice, participants engaged in the three real trials, during which feedback was not given. All participants were correct on the critical trial. Immediately following the critical trial, participants were asked two questions across two separate screens. The first was “Did you notice anything odd on the screen which was not there on the previous trials? If so, please tell me what you saw, with as much detail as you can. If you did not notice anything, that’s OK—please just type ‘no.’” Participants inputted their response to this question via a text box. After this, they were told on a new screen “On this last trial an image was presented in the middle of the screen, between the words. Even if you cannot recall very much about what you saw, can you guess whether it was an image of a living thing or a non-living thing?” Participants chose between two response buttons labeled “Living” and “Non-Living.” The entire session took approximately 6 min.

### 3.2. Results

All spontaneous responses were evaluated by the first and third author. Spontaneous identifications were counted if the participant correctly named the target (e.g., “camel”), or if a highly similar entity was named (e.g., “a goat” for the deer, “skateboard” for the sled), or a higher level name for the entity (most commonly this was “bug” for the insects, and “animal” for the raccoon). One participant said “bird” for the beetle, and we elected not to count this.

Overall, mammals were noticed a little over half of the time, at 58%, which was a significantly higher rate than that of the insects at 35%, test of two proportions *z* = 2.40, *p* = 0.016, Cohen’s *h* = 0.46, and the inanimates at 21%, *z* = 4.07, *p* < 0.001, Cohen’s *h* = 0.77. The identification rate for the insects was only marginally higher than that of the inanimates, *z* = 1.66, *p* = 0.097, Cohen’s *h* = 0.31.

If a participant did not notice anything spontaneously, or noticed something but could not correctly identify it (e.g., “I saw a picture but I do not know what”), their guessing data—the second question—were eligible for analysis. This yielded sample sizes of *n* = 24 in the mammal condition, *n* = 35 in the insect condition, and *n* = 48 in the inanimate condition. For these participants, those who were shown a mammal guessed correctly that it was a living thing 79% of the time, which was significantly greater than chance, goodness of fit *χ*^2^(1) = 8.17, *p* = 0.004, and those who were shown an inanimate guessed correctly that it was a non-living thing 69% of the time, which was also significantly greater than chance, *χ*^2^(1) = 6.75, *p* = 0.009. However, those who were shown an insect guessed correctly only 63% of the time, which was not significantly greater than chance, *χ*^2^(1) = 2.31, *p* > 0.12.

### 3.3. Replication

Although mammals were clearly noticed at higher rates than inanimates, insects were only noticed at slightly higher rates, and the difference was not statistically significant. These data supported our predictions. However, as the difference with insects was marginally significant, we elected to conduct a replication of Experiment 2 with a different sample, in order to see if the same results would hold.

This sample was collected approximately 4 months after the original sample, and was an international sample of *N* = 179 from Prolific (www.prolific.co; 82 male). Self-reported race of this sample was: White (*n* = 122), Black (*n* = 25), Hispanic (*n* = 9), East Asian (*n* = 9), South Asian (*n* = 5), mixed (*n* = 4), unreported (*n* = 3), South-East Asian (*n* = 1), and Middle Eastern (*n* = 1). The country of residence of these participants was: United Kingdom (*n* = 81), elsewhere in Europe (n = 49), United States (*n* = 29), Canada (*n* = 6), Australia (*n* = 5), Mexico (*n* = 5), Israel (*n* = 3), and Chile (*n* = 1). An additional 7 participants were sampled but dropped, as they did not notice the color word on two or more real trials, with one of these being the critical trial. Approximately equal numbers of participants were assigned to the mammal (*n* = 56), insect (*n* = 62), and inanimate (*n* = 61) conditions.

Noticing rates in this replication were highly similar to the initial experiment. Mammals were noticed at rate of 53%, which was a significantly higher rate than that of the insects at 32%, *z* = 2.23, *p* = 0.026, Cohen’s *h* = 0.42, and the inanimates at 23%, *z* = 3.31, *p* < 0.001, Cohen’s *h* = 0.62. However, with this sample the identification rate for the insects was not significantly higher than that of the inanimates, *z* = 1.15, *p* > 0.25.

For the guessing data, there were sample sizes of *n* = 26 in the mammal condition, *n* = 42 in the insect condition, and *n* = 47 in the inanimate condition. Mammals were identified as living things 73% of the time, which was significantly greater than chance, goodness of fit *χ*^2^(1) = 5.54, *p* = 0.019, and inanimates were identified as non-living things 81% of the time, which was also significantly greater than chance, *χ*^2^(1) = 17.89, *p* < 0.001. Replicating the initial experiment, insects were identified as livings things only 41% of the time, which was not different than chance, *χ*^2^(1) = 1.52, *p* > 0.21.

### 3.4. Omnibus analysis

Despite some differences in sample characteristics, we also ran an omnibus analysis pooling the initial and replication experiments together, since larger sample sizes are directly proportional to determining which percentage differences will be deemed significant with proportional data. In this case. In this case, the total sample sizes were *n* = 113 in the mammal condition, *n* = 116 in the insect condition, and *n* = 122 in the inanimate condition.

Overall, mammals were noticed at 57%, which was a significantly higher rate than that of insects at 35%, test of two proportions *z* = 3.29, *p* = 0.001, Cohen’s *h* = 0.45, and inanimates at 22%, *z* = 5.52, *p* < 0.001, Cohen’s *h* = 0.74. In this analysis, the identification rate for the insects was also significantly higher than that of the inanimates, *z* = 2.32, *p* = 0.020, Cohen’s *h* = 0.29. Note that the effect size for the mammal advantage over inanimates was larger than the effect size insect advantage. Table 1 displays the noticing rates for all entities from the omnibus analysis.

**Table 1:** Detection rates for each entity in Experiments 2 and 3.

Experiment 2: Omnibus data			Experiment 3	
Mammal	Insect	Inanimate	Bird	Herpetofauna
Fox: 69%	Bee: 50%	Helicopter: 33%	Eagle: 70%	Frog: 62%
Camel: 58%	Beetle: 46%	Flag: 24%	Chicken: 62%	Snake: 52%
Raccoon: 55%	Grasshopper: 33%	Kite: 17%	Ostrich: 55%	Turtle: 46%
Deer: 50%	Ant: 32%	Whistle: 17%	Owl: 38%	Alligator: 43%
Lion: 47%	Butterfly: 13%	Sled: 16%		

For the guessing data, sample sizes were *n* = 48 in the mammal condition, *n* = 75 in the insect condition, and *n* = 95 in the inanimate condition. For these participants, those who were shown a mammal guessed correctly that it was a living thing 69% of the time, which was significantly greater than chance, goodness of fit *χ*^2^(1) = 7.37, *p* = 0.007, and those who were shown an inanimate guessed correctly that it was a non-living thing 75% of the time, which was also significantly greater than chance, *χ*^2^(1) = 23.25, *p* < 0.001. However, those who were shown an insect guessed correctly only 49% of the time, which was no different than chance, *χ*^2^(1) = 0.01, *p* > 0.90.

### 3.5. Discussion

The data from Experiment 2 show a clear divide in participants’ ability to spontaneously detect an unexpected image of a mammal, an insect, or an inanimate, and replicate the basic finding from Experiment 1. Participants noticed the mammal about half of the time, while they only noticed insects about a third of the time, and inaninmates about a fifth of the time. With the very large sample from the omnibus analysis, we have a reliable estimate that insect detection was about 13% higher than inanimate detection, which is a considerably smaller advantage in comparison to the mammals at 35%. This decreased strength of an animate advantage for insects is likely correlated with the neural representation of insects in the brain, which are represented in a network overlapping with inanimate tools (Sha et al., 2015). This distinction may be driven via the perceived animacy of the entity, which is higher for mammals in comparison to insects (Connolly et al., 2012). An alternative explanation might be that the mammals were rated more highly, on average, in danger or usefulness than the insects and inanimates (e.g., Wurm, 2007). We do not have measures of these dimensions for these image stimuli, but future researchers could incorporate this into their design.

These findings also generally replicate the results of Calvillo and Jackson (2014), who compared animates to inanimates in a very similar inattentional blindness task, but with an additional manipulation of working memory. While they showed a clear advantage for animates, half of their animate stimuli were humans, and another 40% were non-human mammals. Our rates of mammal detection are in the ballpark for their data, but we used a different number of words in the word task (4 vs. their 3 and 6), so the rates are not easily comparable. It may of interest in future studies to compare humans and non-human mammals to each other, in order to see whether humans have an advantage in detection (e.g., Bonatti et al., 2002).

It is also noteworthy that broad categories of living vs. non-living things were accessible to our participants in a partially conscious manner, but the former of these was only possible when the target was a mammal, and not when it was an insect. These results are reminiscent of Wu et al. (2015), who showed that the visual system can rapidly assess the animate vs. inanimate distinction in as short as 120 ms. However, they largely used mammals for their animate stimuli, and our results indicate that insects are not rapidly assessed in the same fashion. We will save further discussion of this issue for the general discussion.

### 3.6. Experiment 3

In Experiment 3, we investigated the detection rates of birds and herpetofauna (reptiles and amphibians) using the same methods as Experiment 2, in order to get further clarity on potential hierarchical differences in animate status. Possidónio et al. (2019) found that people rate birds as having both a higher capacity for cognition and being more similar to humans in comparison to herpetofauna. We hypothesized that an animate’s ability to capture attention would be related to observer’s perception of its animate status, and thus predicted that birds would be recognized more readily than the herpetofauna, but that both would be recognized at rates lower than the mammals from Experiment 1.

### 3.7. Method

#### 3.7.1. Participants

Participants for Experiment 3 were 173 University of Regina undergraduate students (29 male, 2 non-binary), who earned partial course credit for their participation. We aimed for a higher sample size in this experiment than in Experiment 2, as we did not anticipate as large a percentage difference between these animal types as between mammals and insects. Self-reported race of this sample was: White (*n* = 106), South-East Asian (*n* = 21), Black (*n* = 17), South Asian (*n* = 17), Indigenous (*n* = 6), mixed (*n* = 3), West Asian (*n* = 2), and Arab (*n* = 1). An additional 11 people were sampled but dropped, due to missing the color word on 2/3 real trials, with one of these being the critical trial. Approximately equal numbers of participants were assigned to the bird (*n* = 86) and herpetofauna (*n* = 87) conditions.

#### 3.7.2. Stimuli

The target stimuli for this experiment included four birds (chicken, eagle, ostrich, owl) and four herpetofauna (alligator, frog, snake, turtle), taken from Snodgrass and Vanderwort (1980). We only selected 4 animals for each group as we were again constrained by the available animals in the set, and there were only 3 reptiles and 1 frog. Four birds were selected following this, and as in Experiment 2, all of these entities had roughly similar scores for familiarity and image agreement, in the middle range for both measures (Snodgrass and Vanderwort, 1980). They can be viewed at https://osf.io/b2zpf/?view_only=4129537fbef14d018b52e90e6a4c55c1.

#### 3.7.3. Design and procedure

The design and procedure was identical to Experiment 2.

### 3.8. Results

Spontaneous responses were evaluated by the first and fourth author, in the same manner as Experiment 2. One participant reported “a cat in sunglasses” for the owl, and this was not counted.

Overall, birds were noticed about half of the time, at 56%, which was not statistically different from the rate of the herpetofauna at 51%, *z* = 0.69, *p* > 0.49. Although statistical comparisons are not appropriate given that these are different samples, it is clear that these detection rates are extremely similar to the mammal detection rates in Experiment 2. Table 1 displays the noticing rates for each animal.

The sample sizes for those participants who failed to spontaneously identify the image were *n* = 38 in the bird condition and *n* = 43 in the herpetofauna condition. For these participants, those who were shown a bird guessed correctly that it was a living thing only 50% of the time, which was right at chance, and those who were shown herpetofauna correctly guessed that it was a living thing only 44% of the time, which was no different than chance, *χ*^2^(1) = 0.58, *p* > 0.45.

### 3.9. Discussion

The results of Experiment 3 further indicate a role for perceived animacy in animate detection. On the one hand—and in contrast to our prediction—the spontaneous detection rates for the birds and herpetofauna were quite similar to the mammals from Experiment 2, with about half of participants noticing these animals. This shows a fairly clear advantage for these three animal types over inanimates. On the other hand—and in partial support of our prediction—those who failed to notice the image explicitly were no better than chance at guessing whether these animals were living things, while those who viewed mammals in Experiment 2 could do so. This result supports our hypothesis that an animate’s ability to capture attention may be related to its perceived animacy, with birds and herpetofauna ranking slightly lower than mammals, but above insects, on such a hierarchy.

## 4. General discussion

The present findings demonstrate that not all animals are equal in the mind’s eye, unlike what New et al. (2007) originally theorized. Experiment 1 demonstrated that mammals are detected more rapidly than a variety of non-mammals, who did not appear to have an animate advantage at all. This result does not support the idea animate monitoring is applied broadly across all animates. Experiments 2 and 3 painted a more nuanced picture of this initial finding: mammals were very clearly noticed more easily in the context of inattentional blindness in comparison to insects, who were noticed at a much lower, but somewhat higher, rates above inanimates. Birds, reptiles, and amphibians appear to also be noticed at higher rates than inanimates, but even so appear to be processed in a slightly more fragile manner in comparison to mammals, sharing poor implicit recognition as living things along with insects. Taken together, these results suggest that animate monitoring does not operate in an all-or-nothing fashion, and instead may operate in a more graded fashion, potentially as it relates to the animate status of the entities in the observer’s attentional window.

To be clear, the present results certainly do not argue for a rejection of New et al. (2007) original hypothesis. Instead, they suggest there may be limits on (1) how broadly the effect applies across the animal kingdom, and (2) the relative power of a specific animate’s ability to eschew an animate advantage according to context. For example, in Experiment 1 a variety of non-mammals were not detected more quickly than a variety of inanimate objects, while in Experiments 2 and 3 non-mammals were noticed at higher rates than the inanimates (though just barely for insects). This relative difference in the mammal advantage may have been due to task differences. In Experiment 1, attention to the entities was assessed in a competitive fashion (participants were searching for one of either an animate or an inanimate), while in Experiments 2 and 3 the entities themselves were used to evoke observers’ attention. Perhaps because non-mammals are less robustly attended to, they cannot outcompete inanimates in more competitive attentional situations, but can more readily stand out from inanimates when they are encountered unexpectedly. It is also possible that this difference is the result of the differences in the stimuli themselves: relatively unfamiliar, real images of animals were used in Experiment 1, while relatively recognizable line drawings of much more familiar animals (to mostly White Westerners) were used in Experiments 2 and 3. In these latter experiments, this may have given the non-mammals an advantage that they may not normally have in real life, while mammals do not require this advantage to be easily recognized and found. Further research that can directly manipulate the roles of familiarity and type of attention in animate detection could help to tease apart possible explanations for this difference.

Overall, these findings are broadly consonant with neuroimaging research on animals and inanimate tools, which has revealed a continuum of perceived animacy in LOC (Connolly et al., 2012). Sha et al. (2015) demonstrated that there is no neural distinction between animate and inanimate objects in ventral vision. While activation for highly familiar mammals (cats, dogs, and humans) was clearly distinguished from activation for inanimate tools in LOC, activation for less familiar mammals and birds were less clearly differentiated in this respect, and activation for fish and invertebrates clearly overlapped with that for tools. Sha et al. argued that the representation of an animacy continuum in the brain may still be the result of evolutionary pressures, but these shaped visual perception on the basis of the agentive capacity of an animal in the environment, and/or the similarity of the animate in relation to humans. This idea differs from a broad interpretation of animate monitoring that New et al. (2007) originally hypothesized. Future research on differences between types of animates should increase the number of types as well, in order to get more detail on this possible hierarchy. For example, the present research included Mollusca only in Experiment 1, and did not examine fish in any of the experiments.

What is the ultimate nature of these differences, mechanistically? Let us first consider the possible role of surface visual features. In Experiment 1, although our targets did not differ in average luminosity or contrast, they may have differed in mid-level features such as curvilinearity, and this may also have been the case for the line drawings in Experiments 2 and 3. Such a difference would most likely have been present for the mammal/inanimate comparison, and less likely for the mammal/non-mammal comparison. However, considering curvilinearity on its own, a higher degree should have made search more difficult, as Long et al. (2017) found that inanimate texforms were found faster than animate texforms. Further, the results of He and Cheung (2019) indicated that when certain mid-level features are controlled for (i.e., overall shape) an animate advantage still persists. Further investigation into the nature of these broad visual features will certainly be fruitful, but such features do not seem readily poised to explain the present results. On the other hand, visual features *must* be playing some role in the findings, as the different types of animates all share common morphological features which are strongly correlated within their respective categories. Perhaps the parts of mammals are more rapidly encoded or integrated into a whole than the parts of birds and herpetofauna, and perhaps the parts of insects are the most slowly encoded or integrated of all the animates. For insects in particular, perhaps these processes are slower than the rapid assessment of whether an entity is inanimate (Crouzet et al., 2012), and this is why insects were not implicitly recognized as being living things by our participants in Experiment 2.

Another possibility is that non-mammals are not encoded in as robust a manner in working memory as mammals during encoding. This could be related to perceptual differences in parts or overall form, as discussed in the preceding paragraph, but it may also be due to semantic differences between the entities (e.g., mammals as better representatives for the concept “animate”). In Experiment 1, while the presentation stage was seemingly long enough (3 s) to promote sufficient encoding of the entities, there may be differences in the strength or survivability of the representations for mammals in comparison to non-mammals and inanimates (especially in the context of more complex search). It is also possible that encoding of the mammals was superior to the other entities during the search stage itself. Differences in how easily the entities could be recognized in Experiments 2 and 3, both consciously and unconsciously, may also be related to how robustly they can intrude on attention and working memory in the midst of another task. Hagen and Laeng (2017) provided evidence that animates do not appear to be attended to preferentially compared to inanimates in an RSVP task, but do appear to be reported more accurately in such tasks. They similarly argued that this finding is most consistent with either post-attentive perceptual processing, or survivability in short-term memory. The present findings suggest that such processes most readily apply only to mammals, however, and apply to other animals less so as we move down a potential hierarchy of animate status.

More broadly, these findings argue for a theoretical shift in our conceptualization of animate monitoring, but the exact nature of the shift requires further study and investigation. One possibility is that a more accurate term for the effect might be “agentive monitoring.” Evolution may have forged an attentional system in humans that prioritizes entities in the environment that have a relatively high capacity to notice or react the observer themselves. Mammals would subsequently receive the greater prioritization than birds, reptiles, or insects. Perhaps stronger “agentivity” in an animate boost processing more than just animacy itself. Blunt and VanArsdall (2021) found animate imagery and featural animacy had additive effects on word memory; Perhaps these concepts relate to the different animates used in the present research. A distinct possibility is that evolution may have shaped a system which prioritizes humans, and that mammals get prioritized over birds in that they are perceived to be more similar to humans, and thus get a little boost in processing as a result (see Ritchie et al., 2021, for a related discussion of the organization of LOC). A third possibility is that animate monitoring in adults is the result of experience, and not evolution. Mammals may have more robust representations in working memory because they are encountered more frequently than non-mammals, or interacted with in a deeper way over the course of development.

We thus see two important directions for future research on animate monitoring. First, there is a need for a more systematic investigations pitting the detection of various classes of animals against one another. This will provide more clarity on the precise nature of a potential hierarchy of animals, which may map onto a perceived animacy/agentive continuum. Second, there is a need for further explorations into the nature of animate monitoring in early development (i.e., Loucks et al., 2020). Such investigations are critical in distinguishing between evolutionary vs. experiential origins of animate monitoring observed in adulthood.

## Data availability statement

The original contributions presented in the study are included in the article/supplementary material, further inquiries can be directed to the corresponding author.

## Ethics statement

The studies involving human participants were reviewed and approved by the University of Regina Research Ethics Board. The patients/participants provided their written informed consent to participate in this study.

## Author contributions

JL, BR, RG, and SF contributed to conception and design of the studies. BR developed stimuli and designed the methodology for Experiment 1. JL performed the statistical analysis and wrote the first draft of the manuscript. BR, RG, and SF wrote sections of the manuscript. JL and BR contributed to manuscript revision. All authors read and approved the submitted version.

## Funding

This work was funded by a Natural Sciences and Engineering Research Council of Canada Discovery Grant (awarded to JL). Experiment 1 was also developed while BR held a MITACS Globalink Internship to work in JL’s lab.

## Conflict of interest

The authors declare that the research was conducted in the absence of any commercial or financial relationships that could be construed as a potential conflict of interest.

## Publisher’s note

All claims expressed in this article are solely those of the authors and do not necessarily represent those of their affiliated organizations, or those of the publisher, the editors and the reviewers. Any product that may be evaluated in this article, or claim that may be made by its manufacturer, is not guaranteed or endorsed by the publisher.

## References

[ref1] Anwyl-Irvine A. L. Massonnié J. Flitton A. Kirkham N. Evershed J. K. (2020). Gorilla in our midst: an online behavioral experiment builder. Behav. Res. Methods 52, 388–407. doi: 10.3758/s13428-019-01237-x, PMID: 31016684 PMC7005094

[ref2] Blunt J. R. VanArsdall J. E. (2021). Animacy and animate imagery improve retention in the method of loci among novice users. Mem. Cogn. 49, 1360–1369. doi: 10.3758/s13421-021-01175-0, PMID: 33837512

[ref3] Bonatti L. Frot E. Zangl R. Mehler J. (2002). The human first hypothesis: identification of conspecifics and individuation of objects in the young infant. Cogn. Psychol. 44, 388–426. doi: 10.1006/cogp.2002.0779, PMID: 12018939

[ref4] Calvillo D. P. Jackson R. E. (2014). Animacy, perceptual load, and inattentional blindness. Psychon. Bull. Rev. 21, 670–675. doi: 10.3758/s13423-013-0543-8, PMID: 24197657

[ref5] Connolly A. C. Guntupalli J. S. Gors J. Hanke M. Halchenko Y. O. Wu Y.-C.. (2012). The representation of biological classes in the human brain. J. Neurosci. 32, 2608–2618. doi: 10.1523/JNEUROSCI.5547-11.2012, PMID: 22357845 PMC3532035

[ref6] Crouzet S. M. Joubert O. R. Thorpe S. J. Fabre-Thorpe M. (2012). Animal detection precedes access to scene category. PLoS One 7:e51471. doi: 10.1371/journal.pone.0051471, PMID: 23251545 PMC3518465

[ref7] Eddy T. J. Gallup G. G. J. Povinelli D. J. (1993). Attribution of cognitive states to animals: anthropomorphism in comparative perspective. J. Soc. Issues 49, 87–101. doi: 10.1111/j.1540-4560.1993.tb00910.x

[ref8] Guerrero G. Calvillo D. P. (2016). Animacy increases second target reporting in a rapid serial visual presentation task. Psychon. Bull. Rev. 23, 1832–1838. doi: 10.3758/s13423-016-1040-7, PMID: 27112561

[ref9] Hagen T. Espeseth T. Laeng B. (2018). Chasing animals with split attention: are animals prioritized in visual tracking? i-Perception 9, 1–35. doi: 10.1177/2041669518795932, PMID: 30202509 PMC6124190

[ref10] Hagen T. Laeng B. (2016). The change detection advantage for animals: an effect of ancestral priorities or progeny of experimental design? i-Perception 7, 1–17. doi: 10.1177/2041669516651366, PMID: 27433331 PMC4934668

[ref11] Hagen T. Laeng B. (2017). Animals do not induce or reduce attentional blinking, but they are reported more accurately in a rapid serial visual presentation task. i-Perception 8, 1–35. doi: 10.1177/2041669517735542, PMID: 29085619 PMC5648101

[ref12] He C. Cheung O. S. (2019). Category selectivity for animals and man-made objects: beyond low-and mid-level visual features. J. Vis. 19:22. doi: 10.1167/19.12.22, PMID: 31648308

[ref13] JacksonR. E.CalvilloD. P. (2013). Evolutionary relevance facilitates visual information processing. Evol. Psychol. 11:147470491301100. doi: 10.1177/14747049130110050624184882

[ref14] Kellert S. R. (1993). Values and perceptions of invertebrates. Conserv. Biol. 7, 845–855.

[ref15] Knight A. J. (2008). “Bats, snakes and spiders, oh my!” how aesthetic and negativistic attitudes, and other concepts predict support for species protection. J. Environ. Psychol. 28, 94–103. doi: 10.1016/j.jenvp.2007.10.001

[ref16] Lipp O. V. Derakshan N. Waters A. M. Logies S. (2004). Snakes and cats in the flower bed: fast detection is not specific to pictures of fear-relevant animals. Emotion 4, 233–250. doi: 10.1037/1528-3542.4.3.233, PMID: 15456393

[ref17] LoBue V. DeLoache J. S. (2008). Detecting the snake in the grass: attention to fear-relevant stimuli by adults and young children. Psychol. Sci. 19, 284–289. doi: 10.1111/j.1467-9280.2008.02081.x 18315802

[ref18] Long B. Störmer V. S. Alvarez G. A. (2017). Mid-level perceptual features contain early cues to animacy. J. Vis. 17:20. doi: 10.1167/17.6.20, PMID: 28654965

[ref19] Loucks J. Verrett K. Reise B. (2020). Animates engender robust memory representations in adults and young children. Cognition 201:104284. doi: 10.1016/j.cognition.2020.104284 32276235

[ref20] Macé M. J.-M. Joubert O. R. Nespoulous J.-L. Fabre-Thorpe M. (2009). The time-course of visual categorizations: you spot the animal faster than the bird. PLoS One 4:e5927. doi: 10.1371/journal.pone.0005927, PMID: 19536292 PMC2693927

[ref21] Nairne J. S. VanArsdall J. E. Cogdill M. (2017). Remembering the living: episodic memory is tuned to animacy. Curr. Dir. Psychol. Sci. 26, 22–27. doi: 10.1177/0963721416667711

[ref22] New J. Cosmides L. Tooby J. (2007). Category-specific attention for animals reflects ancestral priorities, not expertise. Proc. Natl. Acad. Sci. U. S. A. 104, 16598–16603. doi: 10.1073/pnas.0703913104, PMID: 17909181 PMC2034212

[ref23] New J. J. German T. C. (2015). Spiders at the cocktail party: an ancestral threat that surmounts inattentional blindness. Evol. Hum. Behav. 36, 165–173. doi: 10.1016/j.evolhumbehav.2014.08.004

[ref24] Nguyen H. B. van Buren B. (in press). May the force be against you: better visual sensitivity to speed changes opposite to gravity. J. Exp. Psychol. Hum. Percept. Perform. 10.1037/xhp0001115 37227857

[ref25] Possidónio C. Graça J. Piazza J. Prada M. (2019). Animal images database: validation of 120 images for human-animal studies. Animals 9:475. doi: 10.3390/ani9080475 31344828 PMC6727086

[ref26] Pratt J. Radulescu P. V. Guo R. M. Abrams R. A. (2010). It’s alive!: animate motion captures visual attention. Psychol. Sci. 21, 1724–1730. doi: 10.1177/0956797610387440 20974713

[ref27] Ritchie J. B. Zeman A. A. Bosmans J. Sun S. Verhaegen K. Op de Beeck H. P. (2021). Untangling the animacy organization of occipitotemporal cortex. J. Neurosci. 41, 7103–7119. doi: 10.1523/JNEUROSCI.2628-20.2021, PMID: 34230104 PMC8372013

[ref28] Sha L. Haxby J. V. Abdi H. Guntupalli J. S. Oosterhof N. N. Halchenko Y. O.. (2015). The Animacy continuum in the human ventral vision pathway. J. Cogn. Neurosci. 27, 665–678. doi: 10.1162/jocn_a_00733, PMID: 25269114

[ref29] Snodgrass J. G. Vanderwort M. (1980). A standardized set of 260 pictures: norms for name agreement, image agreement, familiarity, and visual complexity. J. Exp. Psychol. Hum. Learn. Mem. 6, 174–215. PMID: 7373248 10.1037//0278-7393.6.2.174

[ref30] Tisdell C. Wilson C. Swarna Nantha H. (2006). Public choice of species for the ‘ark’: phylogenetic similarity and preferred wildlife species for survival. J. Nat. Conserv. 14, 97–105. doi: 10.1016/j.jnc.2005.11.001

[ref31] van Buren B. Scholl B. J. (2017). Minds in motion in memory: enhanced spatial memory driven by the perceived animacy of simple shapes. Cognition 163, 87–92. doi: 10.1016/j.cognition.2017.02.006, PMID: 28292667

[ref32] Wichmann F. A. Drewes J. Rosas P. Gegenfurtner K. R. (2010). Animal detection in natural scenes: critical features revisited. J. Vis. 10, 1–27. doi: 10.1167/10.4.6, PMID: 20465326

[ref33] Willenbockel V. Sadr J. Fiset D. Horne G. O. Gosselin F. Tanaka J. W. (2010). Controlling low-level image properties: the SHINE toolbox. Behav. Res. Methods 42, 671–684. doi: 10.3758/BRM.42.3.671, PMID: 20805589

[ref34] Wu C.-T. Crouzet S. M. Thorpe S. J. Fabre-Thorpe M. (2015). At 120 msec you can spot the animal but you don’t yet know it’s a dog. J. Cogn. Neurosci. 27, 141–149. doi: 10.1162/jocn_a_00701, PMID: 25208739

[ref35] Wurm L. H. (2007). Danger and usefulness: an alternative framework for understanding rapid evaluation effects in perception? Psychon. Bull. Rev. 14, 1218–1225. doi: 10.3758/BF03193116, PMID: 18229500

[ref36] Yang J. Wang A. Yan M. Zhu Z. Chen C. Wang Y. (2012). Distinct processing for pictures of animals and objects: evidence from eye movements. Emotion 12, 540–551. doi: 10.1037/a0026848 22251055

[ref37] Zachariou V. Del Giacco A. C. Ungerleider L. G. Yue X. (2018). Bottom-up processing of curvilinear visual features is sufficient for animate/inanimate object categorization. J. Vis. 18:3. doi: 10.1167/18.12.3, PMID: 30458511 PMC6222807

